# Epigenetic reader chromodomain as a potential therapeutic target

**DOI:** 10.1039/d4cb00324a

**Published:** 2025-04-11

**Authors:** Shivangi Sharma, J. Trae Hampton, Tatiana G. Kutateladze, Wenshe Ray Liu

**Affiliations:** a Texas A&M Drug Discovery Center and Department of Chemistry, Texas A&M University College Station TX 77843 USA wsliu2007@tamu.edu; b Department of Pharmacology, University of Colorado School of Medicine Aurora CO 80045 USA tatiana.kutateladze@cuanschutz.edu; c Institute of Biosciences and Technology and Department of Translational Medical Sciences, College of Medicine, Texas A&M University Houston TX 77030 USA; d Department of Biochemistry and Biophysics, Texas A&M University College Station TX 77843 USA; e Department of Cell Biology and Genetics, College of Medicine, Texas A&M University College Station TX 77843 USA; f Department of Pharmaceutical Sciences, Texas A&M University College Station TX 77843 USA

## Abstract

Epigenetic mechanisms involve cooperative actions of enzymes that produce or remove post-translational modifications in histones and ‘readers’, the protein domains that bind these modifications. Methylation of lysine residues represents one of the most common modifications and is recognized by a family of chromodomains. Chromodomain containing proteins are implicated in transcriptional regulation and chromatin remodeling, and aberrant functions of these proteins are linked to human diseases, such as cancer, neurodegenerative disorders and developmental abnormalities. In this work, we review biological and pathological activities of chromodomains, highlighting their potential as prognostic biomarkers and their attractiveness as therapeutic targets. In the past few years, significant progress has been made in the development of chromodomain inhibitors, however sequence similarity within this family of readers presents challenges in designing selective probes. We describe recent advances and new strategies that are employed to overcome these challenges, including structure-based drug design, high-throughput screening, the use of peptide and DNA encoded libraries, and summarize research underscoring the benefit of targeting chromodomains to combat diseases.

## Abstract 2

Chromodomains are epigenetic readers for histone methylation. Their selective targeting by small molecule or peptide inhibitors may serve as a novel way for disease intervention.

## Graphical abstract

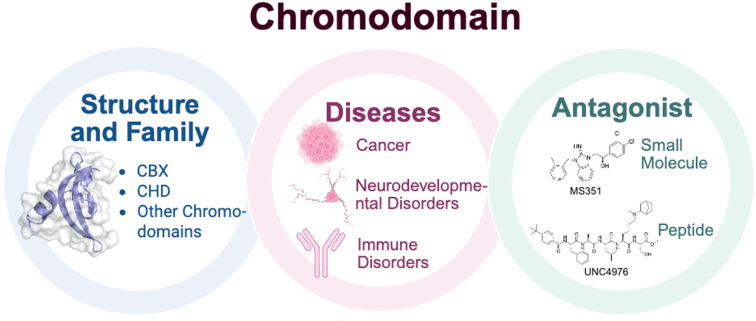

## 1. Introduction

In eukaryotic cells, DNA is wrapped around histone proteins to form the nucleosome, a repeating unit of chromatin. Both DNA and histones undergo covalent modifications, known as epigenetic marks.^1–3^ These marks include generally reversible post translational modifications (PTMs) in the tails of histones and DNA methylation. The epigenetic marks are deposited by the enzymes named ‘writers’, removed by the enzymes named ‘erasers’, and recognized by the protein domains, the so called ‘readers’. Aberrant functions of these epigenetic players are often linked to diseases, such as cancer and neurodegenerative and developmental abnormalities.^4–6^ In depth understanding of the physiological and pathological activities of the components of the epigenetic machinery can lead to new pharmacological approaches to battle these diseases. Among fundamental PTMs recognized by readers are methylation and acetylation of specific amino acids in histones. For example, plant homeodomain (PHD) fingers, chromodomains and other Royal family modules bind methylated lysine (methyllysine) residues, whereas bromodomains, double PHD fingers and YEATS domains bind acetylated lysine (acetyllysine) residues.^7–9^

Bromodomain was identified as the primary reader and is currently the most thoroughly characterized. Despite having little sequence similarity, they share a highly conserved four-helix bundle structure.^10^ It folds into a four-helix bundle that creates a deep, hydrophobic, and therefore druggable, binding pocket of acetyllysine. Recent successful applications of bromodomain inhibitors for the treatment of cancer have opened a new avenue in the development of epi-based therapeutics, including development of inhibitors for chromodomains, which were identified as first readers capable of binding to methyllysine PTMs.^11–13^ A wealth of cellular, mechanistic and clinical studies suggests that inhibition of chromodomains represents an attractive therapeutic strategy. Even minimal dysregulation or mutations in chromodomain proteins have been shown to be associated with a wide range of diseases, particularly cancer. Evaluated by Dscore, which accounts for volume, enclosure and hydrophobicity of binding pockets, chromodomain was found to be most druggable among methyllysine readers.^14^ Still, targeting proteins from this family are challenging. Chromodomains have shallow and hydrophobic binding pockets, offering limited surface area for small molecule interactions. The hydrophobic nature of the pocket also limits the chemical diversity of potential inhibitors. Chromodomains and methyl lysine interact *via* weak, non-covalent interactions, making it challenging to design potent inhibitors. Additionally, the structural similarity of the methyl-lysine binding pocket across various chromodomains and other reader proteins (*e.g.*, Tudor and PWWP domains) makes it hard to develop selective inhibitors, increasing the risk of off-target effects. Many potential chromodomain inhibitors are peptide-based or large molecules, resulting in poor cell permeability and metabolic instability. Furthermore, even when potent inhibitors are identified, their pharmacokinetic properties often hinder their clinical application. In this review we outline biological activities of chromodomain subfamilies and their relationship to diseases, summarize progress in the development of chromodomain inhibitors and discuss the feasibility of discoveries of more potent and selective chemical probes.

## 2. Chromodomains

Chromodomain was originally identified in the HP1 (heterochromatin protein 1) and polycomb proteins, and since both were linked to gene silencing through a chromatin-based mechanism, the homologous region was named Chromatin Organization Modifier or chromodomain.^15^ Chromodomain is a small ∼50-amino acid module which is present in ∼30 mammalian proteins and belongs to the structurally related Royal family of domains.^16^ The Royal family domains are characterized by a barrel-like fold, also known as the Tudor barrel fold, and are predominantly readers of methylated lysine. The Royal family is classified into subfamilies based on additional structural features, with each subfamily showing selectivity toward particular methyllysine PTM. These include the subfamilies of Tudor domains, proline–tryptophan–tryptophan–proline (PWWP) domains, malignant brain tumor (MBT) domains and chromodomains. Based on their structure and mechanisms of action chromodomain-containing proteins are divided into several subgroups, such as canonical chromobox (CBX), chromo-ATPase/helicase-DNA-binding (CHD) and noncanonical.^17,18^

The structure of chromodomain consists of three anti-parallel β-strands and an α-helix. Chromodomains with higher sequence homology to HP1 selectively recognize and interact with conserved methylated-lysine motifs (ARKS) originally found in H3 tail at H3K9 and H3K27 sites, or the same motif present in different proteins. The active site features a hydrophobic cage formed by aromatic residues (tyrosine, tryptophan, phenylalanine), which interact with the methyl groups through cation–π interactions, stabilizing the binding.^18^ Chromodomains that recognize the methyl lysine mark on N-terminal histone tails, play key role in gene regulation and chromatin remodeling. Upon binding, chromodomains recruit chromatin-modifying complexes like polycomb repressive complex 1 and 2 – PRC1/PRC2. These complexes prevent the access of transcription factors thereby resulting in gene silencing. Chromodomains belonging to CBX family are part of these complexes. Conversely, some chromodomains, like CHD1, interact with activating complexes to promote transcription activation.^19^

### 2.1. CBX chromodomains

**Fig. 1:**
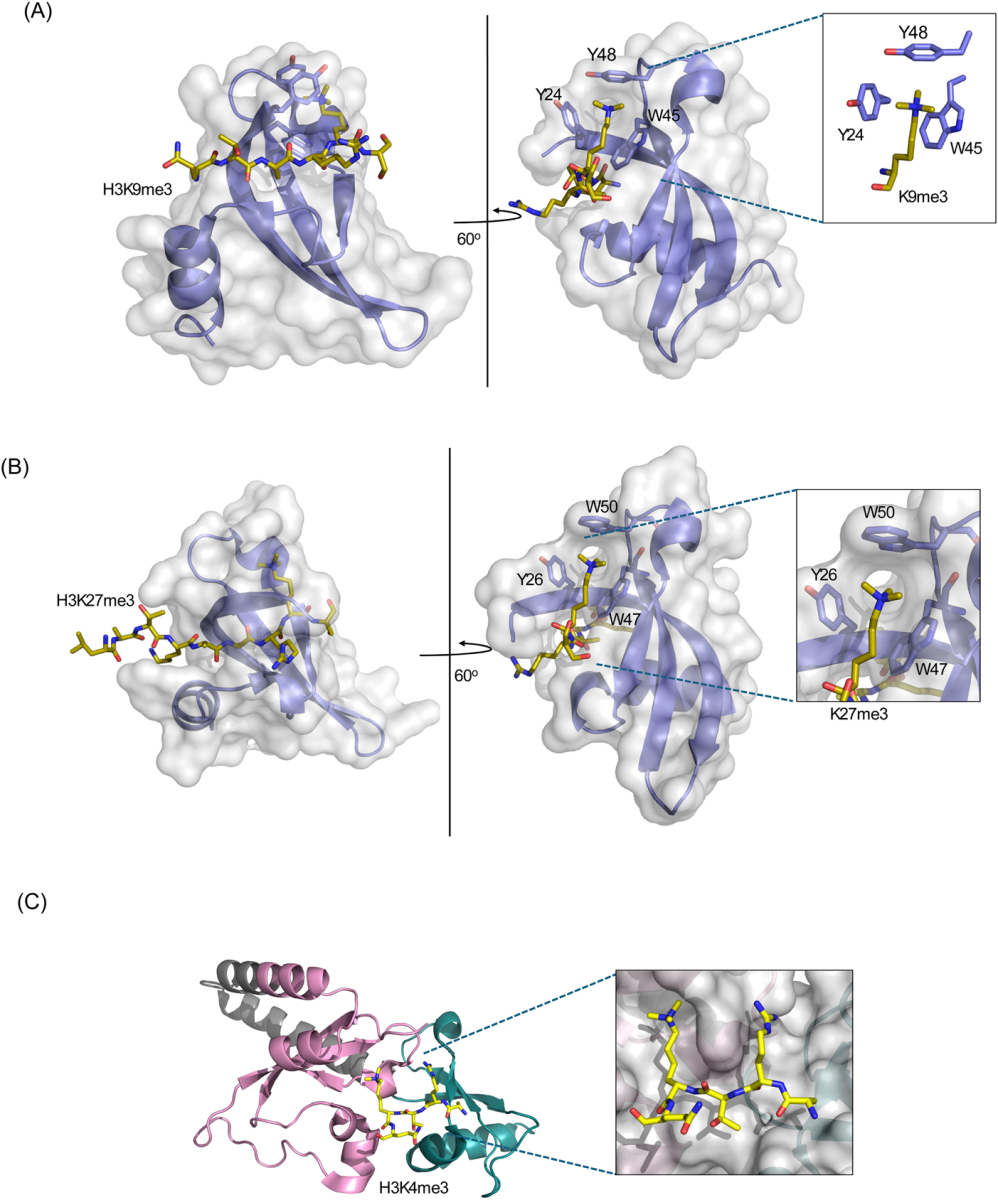
(A) Crystal structure of the Drosophila HP1α chromodomain in complex with H3K9me3 peptide (PDB: 1KNE) with the aromatic cage shown on the right. (B) Crystal structure of the Drosophila PcG in complex with H3K27me3 (PDB: 1PDQ) with the aromatic cage shown on the right. (C) Crystal structure of the tandem chromodomains of CHD1 in complex with H3K4me3 (PDB: 2B2W) (pink, chromodomain 1; grey, linker; cyan, chromodomain 2).

Five polycomb (Pc) proteins – CBX2, CBX4, CBX6, CBX7, CBX8 and three variants of HP1 – HP1α (CBX5), HP1β (CBX1) and HP1γ (CBX3) were identified in mammals with their chromodomains sharing over 60% of sequence similarity.^15^ While HP1 chromodomains recognize di- and trimethylated lysine 9 of histone H3 (H3K9me2/3), Pc/CBX chromodomains recognize di- and trimethylated lysine 27 of histone H3 (H3K27me2/3),^11,20^ but all utilize the same conserved mechanism to engage with the methyllysine substrates. This mechanism involves the insertion of histone tail, which is in an extended conformation, between β-strands of chromodomain and the enclosure of methyllysine within the aromatic cage of chromodomain.^21–24^ The aromatic cage residues and the methylammonium group of lysine are involved in the cation–π interactions that are required for the formation of the complex (Fig. 1). Additionally, electrostatic and polar contacts play an important role in the formation of HP1 chromodomains’ complexes, whereas hydrophobic contacts stabilize the polycomb chromodomains’ complexes.^21,24–26^

Aberrant expression and dysregulated functions of the CBX chromodomain containing proteins are directly linked to the development and progression of cancer.^27,28^ Particularly, the expression of all or some of the CBX1/2/3/4/5/8 members was found to be upregulated in most malignancies, including glioma,^29,30^ pancreatic adenocarcinoma^31^ and breast, gastric and non-small cell lung cancers.^32–34^ In contrast, CBX7 is a tumor suppressor, and its expression level is low in most tumors, such as glioma^29^ and gastric and ovarian cancers.^34,35^ CBX6 expression varies in different cancer types, with low expression observed in glioma,^29,30^ breast and ovarian cancers,^35,36^ but high expression in sarcoma,^37^ head and neck squamous cell carcinoma^38^ and skin cutaneous melanoma.^39^ A recent report has pointed to the direct correlation between the expression of CBXs and cancer progression:^40^ CBXs may be upregulated or downregulated in different types of cancer, and the difference in expression levels is closely related to clinical characteristics like tumor size, clinical grade and stage, relapse, metastasis, vascular invasion, chemoresistance, gene mutation and survival prognosis. In general, CBX1/2/3/4/5/6/8 are tumor promoting factors in most cancers, and CBX7 is tumor-suppressing factor in almost all cancers, thus CBXs could serve as diagnostic and prognostic biomarkers.

### 2.2. CHD chromodomains

There are nine human chromo-ATPase/helicase-DNA-binding (CHD1-9) proteins, and all contain two tandem chromodomains. The chromodomains of CHD1 recognize trimethylated lysine 4 of histone H3 (H3K4me3) *via* a unique mechanism, involving both chromodomains and two aromatic cage residues.^41^ Asymmetric methylation of H3R2 or phosphorylation of H3T3 reduces binding affinity of CHD1 chromodomains and may act as biological binary switches, modulating the CHD1 interaction with H3K4me3-rich chromain.^41^ CHD5 was the first member of this family of ATPases found to have tumor suppressive activity, as it was depleted or inactivated in a wide array of malignancies,^42^ including melanoma,^43^ leukemia,^44^ glioma^45,46^ and lung, prostate and breast cancers.^47–50^ Moreover, loss or inactivation of CHD3/4/5 is associated with chemoresistance, epithelial–mesenchymal transition (EMT), metastasis and poor survival,^45,49,51,52^ and CHD4 deficiency especially was shown to contribute to chemoresistance in BRCA mutant cells.^53^ Much like CHD5, CHD1/2 are found either lost or inactivated in several cancers, but their gain in function also promotes oncogenesis and can be hormone responsive.^54^ CHD6-9 members have been linked to developmental and neurological syndromes, including CHARGE syndrome, schizophrenia, and autism.^55–57^

### 2.3. Other methyllysine binding chromodomains

Chromodomain of MPP8 recognizes methylated H3K9 and belongs to the canonical group of chromodomains.^58,59^ MPP8 associates with the DNA methyltransferase DNMT3A and stimulates tumorigenesis and invasiveness by regulating E-cadherin expression.^59,60^ It promotes proliferation of non-small cell lung cancer, melanoma, and liver cancer cells, and expression levels of MPP8 are found to be upregulated in hepatocellular carcinoma and osteosarcoma.^61^ MPP8 is involved in the regulation of apoptosis of gastric cancer cells and promotes metastasis *via* the p53/Bcl-2 and EMT-related signaling pathways,^62^ whereas loss of MPP8 inhibits development of acute myeloid leukemia.^63^ Another canonical chromodomain is present in the CDY (chromodomain on the Y chromosome) family of enzymes. The chromodomain of CDY is a reader of methylated H3K9 and H3K27 as well as methylated lysines in non-histone proteins.^64–66^ Methylated non-histone substrate, such as retinoic acid-related orphan nuclear receptor α (RORα), is recognized by chromodomain of DCAF1.^58^ Binding of DCAF1 to monomethylated RORα leads to RORα degradation and thus loss of tumor suppressive activity exerted by RORα.

## 3. Antagonists of methyllysine binding chromodomains

Several hundred epigenetic proteins containing methyllysine readers have been identified in the human proteome. These proteins are involved in fundamental chromatin related processes and are implicated in diseases. Therefore, development of chemical small molecule or peptide-based probes has become a priority both for interrogating biological functions of these proteins, as well as facilitating the design of targeted therapeutics.^67–69^ Several inhibitors targeting methyllysine readers MBT and PHD were reported previously, and in 2014 first inhibitors for chromodomains were described.^70^

### 3.1. Inhibitors of CBX chromodomains

**Fig. 2:**
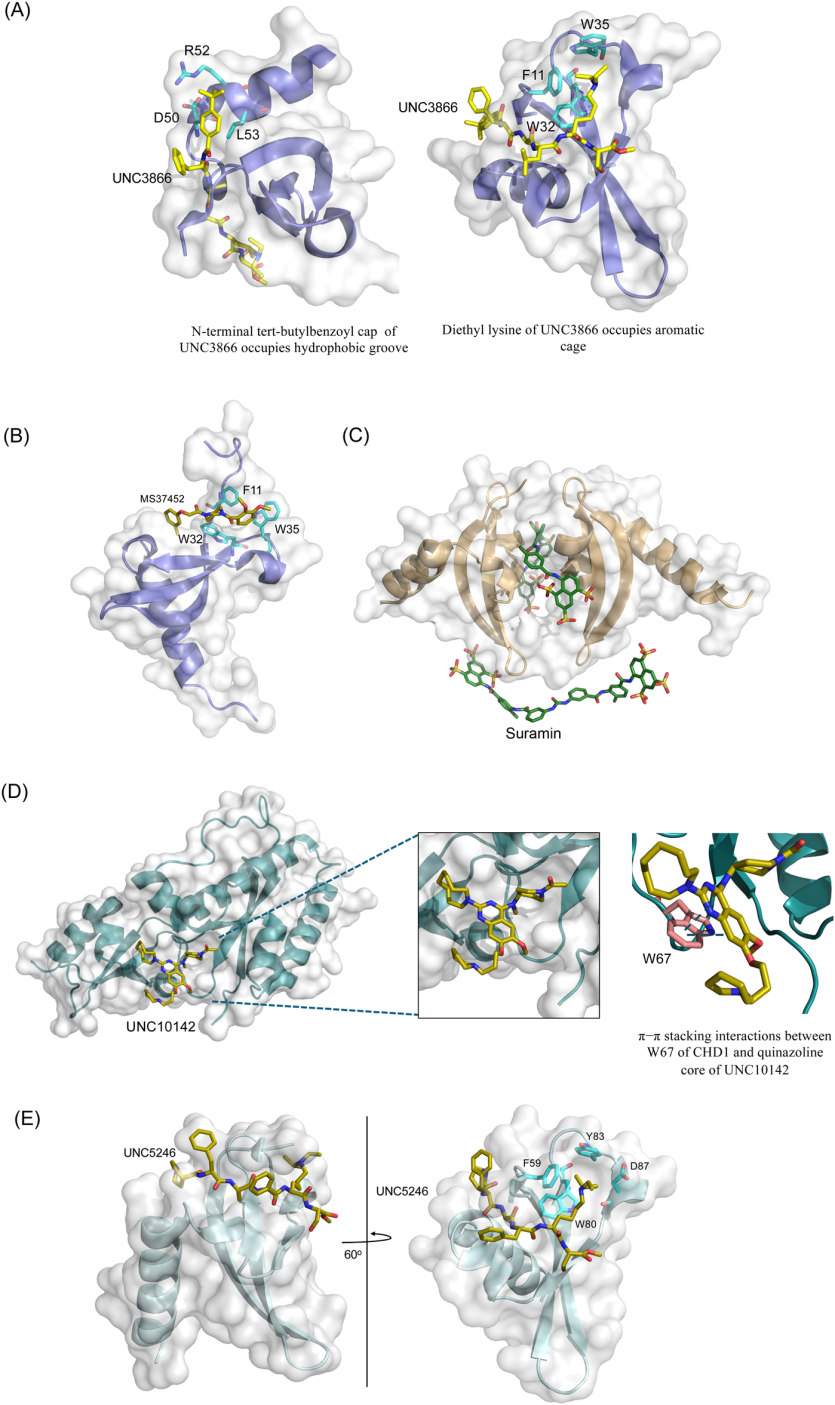
Structures of chromodomains in complex with indicated inhibitors. (A) Chromodomain of CBX7 in complex with inhibitor UNC3866 (PDB code: 5EPJ). (B) Chromodomain of CBX7 in complex with inhibitor MS37452 (PDB code: 4X3T). (C) Chromodomain of CBX7 complexed with suramin (2 : 2 complex of protein : inhibitor) (PDB code: 4X3U). (D) Chromodomain of CHD1 in complex with inhibitor UNC10142 (PDB code: 8UMG). (E) Chromodomain of MPP8 in complex with inhibitor UNC5246 (PDB code: 7M5U).

The first reported chromodomain inhibitors were designed to target CBX7, because the association of CBX7 with H3K27me3 was shown to promote proliferation of cancer cells. The antagonists for CBX7 were obtained through a peptide-driven approach and the structure-based residue substitution of native peptide ligand and tested in antiproliferative assays.^70^ These peptidomimetic inhibitors bind to CBX7 with *K*_D_s ranging from 0.2–4.1 μM as measured by isothermal titration calorimetry (ITC) and fluorescence polarization (FP) assays, with the lead compound 64 showing the *K*_D_ of 200 nM and exhibiting 10-fold selectivity toward CBX7 over CBX8 (Table 1). Further optimization from the lead compound 64 yielded the second generation of peptidomimetic inhibitors though without significant improvement in potency and selectivity.^71^UNC3866, another chemical probe for CBX7 was developed *via* molecular dynamics simulations and SAR studies. UNC3866 does not distinguish between CBX7 and CBX4 and binds to both equally well (*K*_D_ of 97 nM and 94 nM, respectively) (Table 1). The crystal structure of the CBX7–UNC3866 complex shows that the diethyllysine, an analog of methyllysine, occupies the canonical methyllysine-binding aromatic cage, whereas the N-terminal *tert*-butylbenzoyl cap lays in the hydrophobic groove formed by D50, R52 and L53 residues of CBX7^72,73^ (Fig. 2A). A more potent CBX7 inhibitor UNC4976, an analog of UNC3866, was identified in a cellular GFP reporter assay screening. For this assay, mouse embryonic stem cell (mESC) line engineered with a GFP reporter gene controlled by polycomb repressive domain was used. Recruitment of CBX7 to the ZFHD1 (zinc finger homeodomain1) DNA-binding site silenced GFP expression. Cells were treated with UNC4976, UNC3866 and other analogs of UNC3866 for 48 hours, GFP levels were measured using flow cytometry, indicating disruption of CBX7 mediated repression. UNC4976 emerged as the most effective analog, showing ∼14-fold higher potency than UNC3866. The enhanced cellular activity of UNC4976 was attributed to the replacement of the diethyllysine in UNC3866 with *N*ε-methyl- *N*ε-norbornyl-lysine (Table 1). It was proposed that UNC4976 acts as a positive allosteric modulator of CBX7 by promoting non-specific interaction with nucleic acids.^74^

**Table 1:** Overview of inhibitors of chromodomain

Inhibitor	Structure	Target	Potency	Ref.
Compound 64	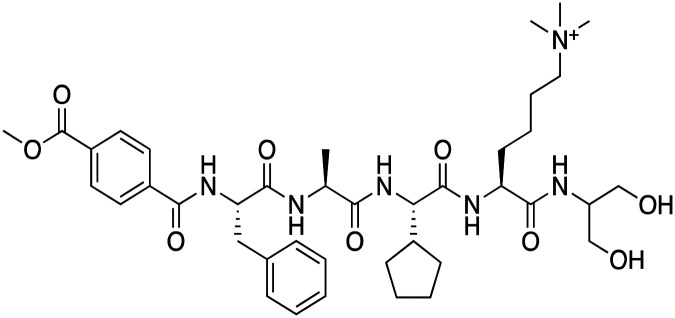	CBX4/7	*K*_D_: 0.2 μM for CBX7 and 0.29 μM for CBX4 (ITC)	70
UNC3866	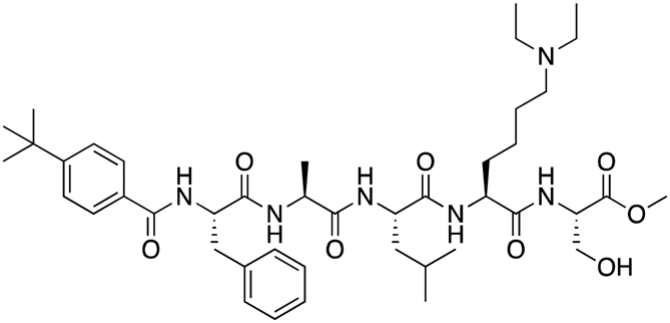	CBX4/7	*K*_D_: 94 nM for CBX4 and 97 nM for CBX7 (ITC); IC50: 66 nM for CBX7 (Alpha Screen)	72 and 73
UNC4976	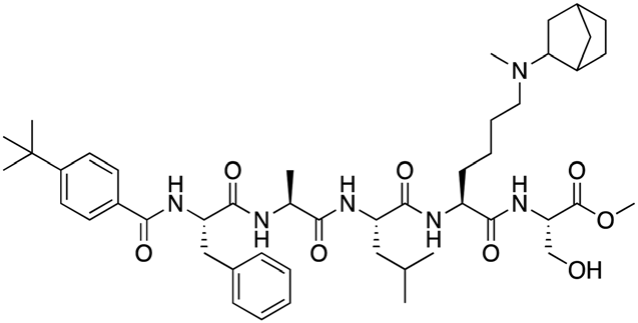	CBX4/7	*K*_D_: 62 nM for CBX4 and 59 nM for CBX7 (ITC)	74
MS37452 (MS452)	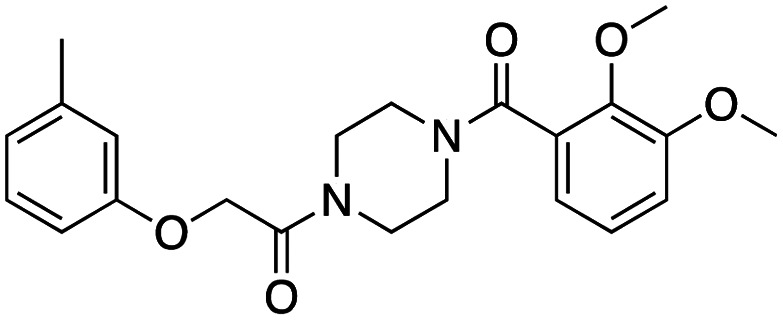	CBX7	*K*_D_: 28.9 μM (NMR); Ki: 43 μM and 55 μM for H3K27me3 and H3K9me3 (FP), respectively	75
Suramin	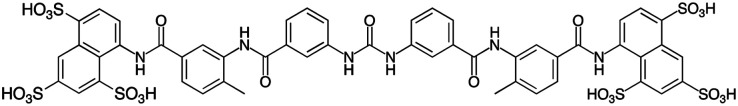	CBX7	IC50: 8.1 μM (FP)	75
MS351	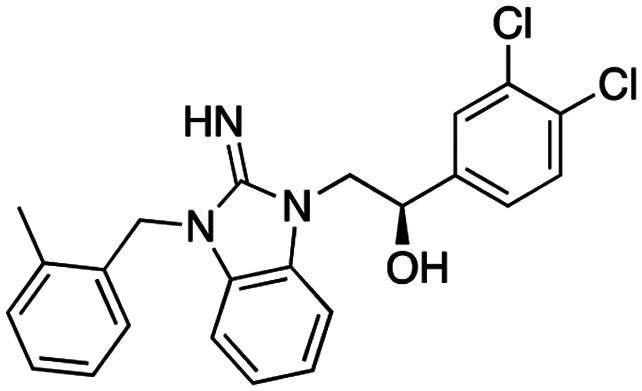	CBX7	*K*_D_: 500 μM (NMR) for free CBX7 and 23.8 μM (FP) for CBX7 in complex with hairpin RNA	76
Compound 5	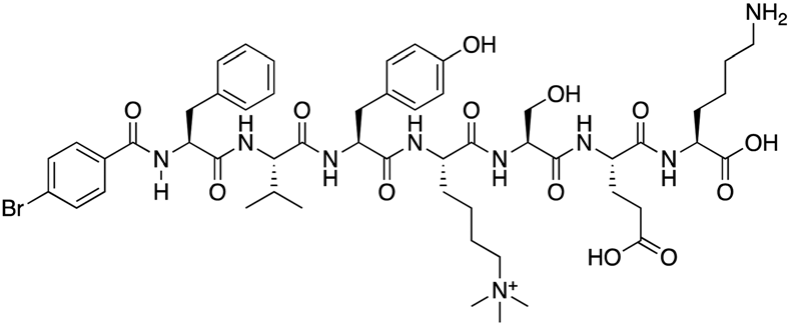	CBX6	*K*_D_: 0.9 μM (FP and SPR) note: >6-fold selectivity from other CBX-chromodomains based on *K*_D_ values (FP)	77
Compound 22	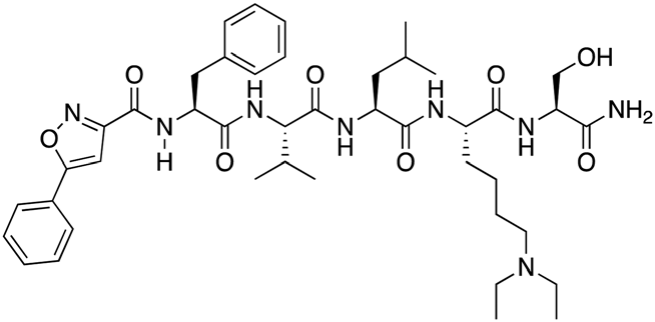	CBX6/8	IC50: 0.2 μM for CBX6/8 (FP)	78
SW2_110A	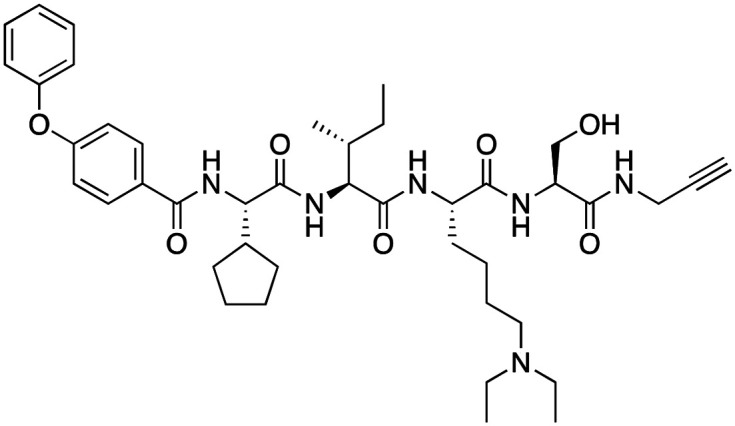	CBX8	*K*_D_: 800 nM (FP) note: >5-fold selectivity from other Pc CBX-chromodomains based on *K*_D_ values	79
UNC7040	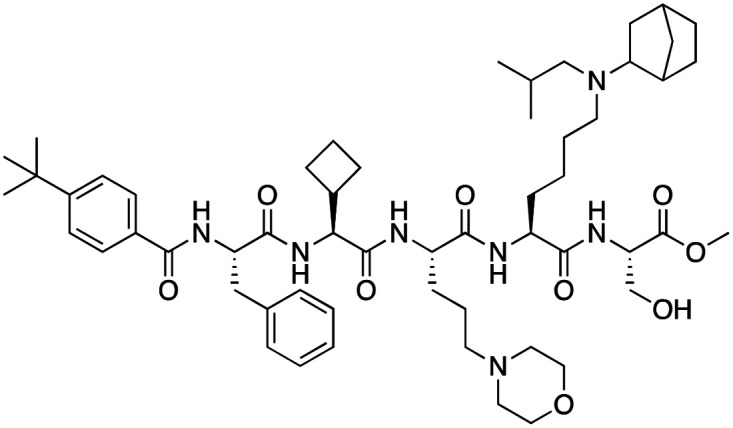	CBX8	*K*_D_: 0.16 μM (SPR); IC50: 0.65 μM (TR-FRET)	80
SW2_152F	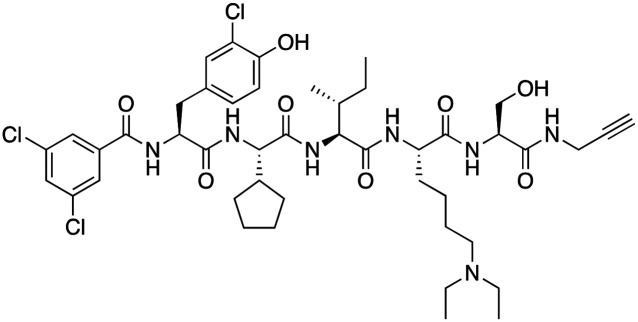	CBX2	*K*_D_: 80 nM (FP); IC50: 2.07 μM (FP) note: >24-fold selectivity from other Pc CBX-chromodomains based on *K*_D_ values	81
Compound 1	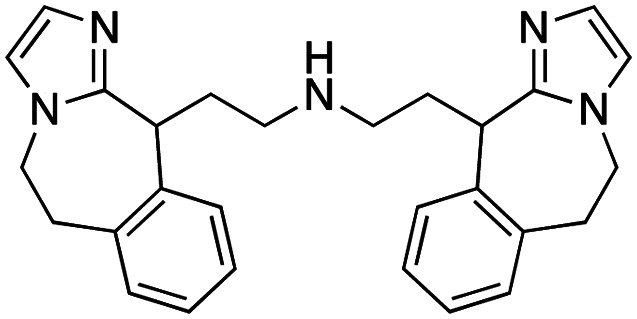	CBX2	*K*_D_: 0.1 μM (SPR); IC50: 1.3 μM (TR-FRET)	82
Compound 2	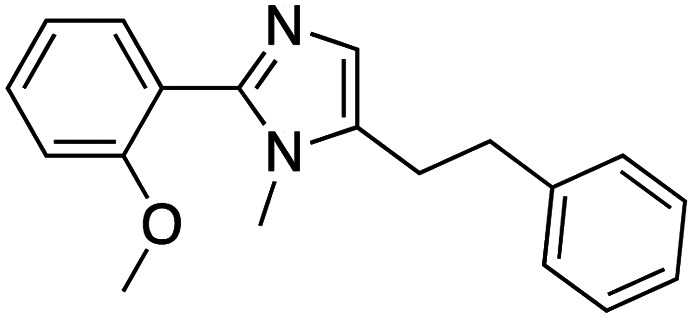	CBX2	*K*_D_: 2.4 μM (SPR); IC50: 0.59 μM (TR-FRET)	82
UNC7560	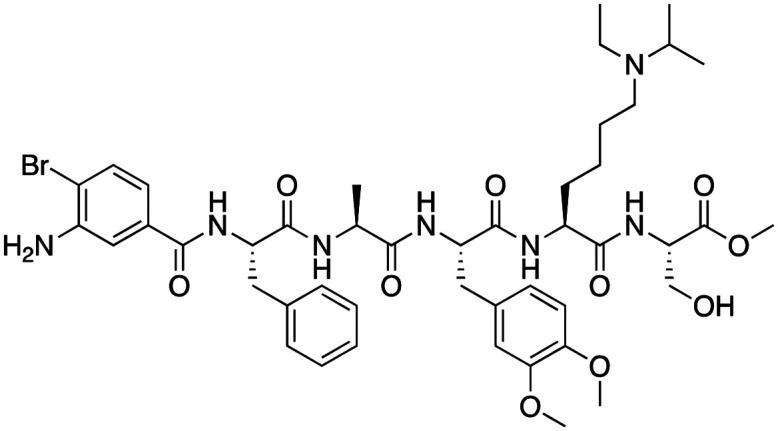	CBX5	*K*_D_: 0.28 μM for CBX5/HP1α, 0.48 μM for CBX1/HP1β, 0.42 μM for CBX3/HP1γ (ITC); IC50: 0.13 μM for CBX5 (TR-FRET)	83
UNC10142	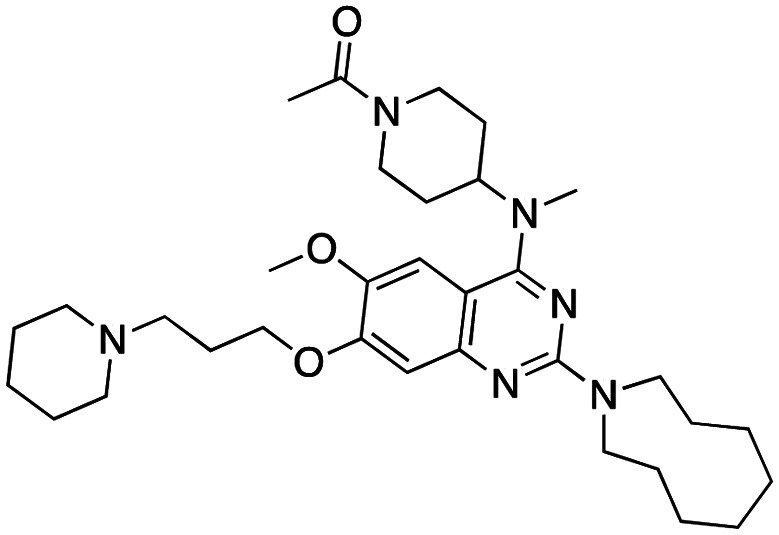	CHD1	IC50: 1.7 μM (TR-FRET) *K*_D_ = 4.3 μM (ITC)	84
UNC4991	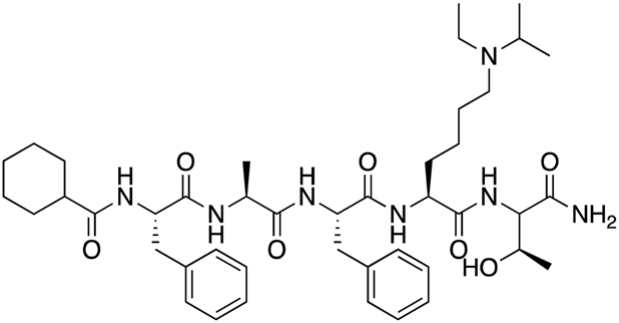	CDYL2/CDYL	*K*_D_: 0.64 and 0.49 μM (ITC) or 0.43 and 1.3 μM (FP) for CDYL2 and CDYL	85
UNC4850	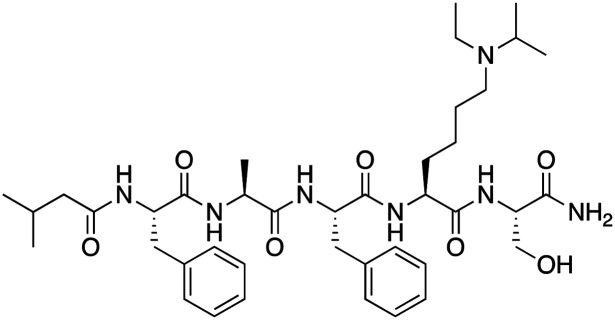	CDYL2/CDYL	*K*_D_: 0.42 μM for CDYL2 and 0.47 μM for CDYL (ITC)	86
UNC5246	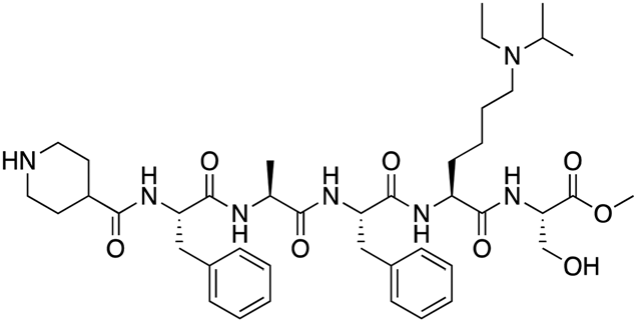	MPP8/CDYL2	*K*_D_: 0.72 μM for MPP8 and 0.17 μM for CDYL2 (ITC); IC50: 0.5 μM for MPP8 and 0.09 μM for CDYL2 (TR-FRET)	87

Small molecule antagonists of CBX7, MS37452 (MS452) and suramin, were identified in fluorescence polarization (FP) based high throughput screening (HTS)^75^ (Table 1). MS37452 shows moderate affinity to other Pc chromodomains (*e.g.*, CBX4) but it is inactive against HP1 chromodomains. Analysis of chromatin immunoprecipitation (ChIP) indicated that MS37452 at the concentration of 250 μM for 2 hours effectively releases the transcriptional suppression of the target gene p16/CDKN2A in PC3 prostate cancer cells *via* disrupting the recruitment of CBX7 to the INK4A/ARF locus.^75^ In the crystal structure of the CBX7–MS37452 complex, the dimethoxybenzene and piperazine rings are bound in the aromatic cage of CBX7, supporting that MS37452 antagonizes methyllysine substrates (Fig. 2B). Interestingly, in the suramin–CBX7 complex two suramin molecules associate with two CBX7 molecules, though we note that suramin is recognized as a promiscuous compound across various screening contexts^75^ (Fig. 2C). Another small-molecule inhibitor of CBX7, MS351, was identified through virtual screening and was confirmed to be an allosteric regulator with cellular potency greater than that of MS37452 (Table 1). MS351 was shown to effectively induce transcriptional derepression of CBX7 target genes.^76^

Compound 5, a selective inhibitor of CBX6,^77^ and compound 22, a dual-activity inhibitor of CBX6/8,^78^ were identified using FP and competitive FP assays from a series of analogs of compound 64 (Table 1). A 900 nM binding affinity of compound 5 to CBX6 was measured by FP assay.^77^ The selective and potent inhibitor of CBX8 was discovered *via* screening DNA-encoded libraries (DELs). SW2_110A binds CBX8 with a *K*_D_ of 800 nM and displays ∼5-fold selectivity for CBX8 over all other CBX paralogs. Using SW2_110A to disrupt the interaction between CBX8 and chromatin, Wang *et al.* show the importance of functional chromodomain of CBX8 in proliferation of MLL-AF9 leukemia cells^79^ (Table 1). UNC7040, a potent positive allosteric modulator (PAM) of CBX8, was developed using the same approach as UNC4976 (Table 1). UNC7040 disrupts the association of CBX8 with H3K27me3-rich chromatin but promotes non-specific interaction of CBX8 with nucleic acids and antiproliferative activity in diffuse large B cell lymphoma and colorectal cancer cell lines.^80^

A core component of the canonical PRC1 complex and the reader of H3K27me3, CBX2, is overexpressed in metastatic neuroendocrine prostate cancer.^88^SW2_152F, a selective CBX2 chromodomain probe was discovered through selections of focused DELs (Table 1). SW2_152F binds to CBX2 with a *K*_D_ of 80 nM and displays 24-1000-fold selectivity for CBX2 over other CBX paralogs. SW2_152F selectively disrupts CBX2's association with chromatin and inhibits the proliferation of LNCaP_NED cells, a subtype of androgen receptor (AR) antagonist-resistant cells derived from the androgen-sensitive prostate cancer cell line LNCaP.^81^ Recently, using nucleosome-based time-resolved fluorescence resonance energy transfer (TR-FRET) screening, compound 1 and compound 2 were identified as moderately potent small-molecule inhibitors of CBX2^82^ (Table 1). UNC7560, a CBX5-targeting ligand with a *K*_D_ of 280 nM (ITC) was found to be modestly selective for HP1 CBXs over PcG CBXs^83^ (Table 1). It has been proposed that larger alkyl substituents in the alkylammonium group of lysine in the CBX5 ligands enhance binding affinity of CBX5, which can open a potential avenue for developing more effective chemical probes targeting CBX5.^89^

### 3.2. Inhibitors of CHD chromodomains

CHD1 is a synthetic lethal target in phosphatase and tensin homologue (PTEN)-deficient cancers.^84^ Although it has been acknowledged as an attractive pharmacological target, no inhibitors or antagonists of CHD1 were reported until 2024. The first-in-class small molecule antagonist of tandem chromodomain CHD1, UNC10142, binds with an IC50 of 1.7 ± 0.2 μM. The UNC10142 binding mechanism derived from the crystal structure of the UNC10142–CHD1 complex appears to be dependent on π–π stacking rather than the cation–π interactions found in complexes with other antagonists (Table 1) (Fig. 2D). UNC10142 selects for CHD1 against a panel of other methyllysine readers, and treatment of PTEN-deficient prostate cancer cells with UNC10142 leads to a dose-dependent reduction in viability, phenocopying genetic loss of CHD1.^84^

### 3.3. Inhibitors of other chromodomains

CDYL2/CDYL are required for normal spermatogenesis and central nervous system development. UNC4991 was identified as selective and potent peptidomimetic inhibitor of CDYL2/CDYL with a >5 fold selectivity toward CDYL2/CDYL over other chromodomains^85^ (Table 1). Further structure-based optimization yielded UNC4850, a sub-micromolar ligand for CDYL2 and CDYL1b with 10-fold selectivity to both over CBX7, likely because it contains an isobutyl group instead of the cyclohexyl group in UNC4991^86^ (Table 1). UNC5246, a peptidomimetic ligand for chromodomain of MPP8, which is part of the HUSH complex, was initially developed using one-bead, one-compound (OBOC) combinatorial screening approach^87^ (Table 1). UNC5246 binds to MPP8 with a *K*_D_ of 0.72 μM and is greater than 70-fold selective for MPP8 over CBX7, however it binds even tighter to CDYL2 (*K*_D_ of 0.17 μM). The structure of the MPP8–UNC5246 complex shows that the ethyl-isopropyl lysine mimetic of UNC5246 occupies the MPP8 aromatic cage, with the isopropyl moiety pointing toward the cage and the ethyl group pointing toward solvent (Fig. 2E).

As summarized, chromodomain inhibitors are primarily peptide-based to effectively mimic the native histone sequences, offer higher binding affinity, and provide better selectivity and versatility compared to small molecules, which face challenges due to the shallow and hydrophobic nature of the chromodomain binding pocket.

## 4. Other development

Display techniques that physically bridge genotypic nucleic acids and phenotypic peptides or small molecules have been widely used in selection-based drug discovery.^90^ Prominent methods in this domain include phage display, mRNA display, and yeast display. Among these, phage display, with its ability to encode protein domains, has been employed to evolve high affinity chromodomains with enhanced detection of histone methylation marks.^91^ Despite their potential, the application of display techniques for identifying chromodomain-targeting molecules has yet to achieve significant success. Liu *et al.* have recently developed a novel phage display approach called phage-assisted, active site-directed ligand evolution (PADLE).^92^ This technique allows genetic incorporation of noncanonical amino acids, specifically posttranslationally modified lysines or their mimics, into phage display libraries. By doing so, PADLE directs displayed peptides toward active sites of epigenetic proteins, including enzymes and readers, for improved selection. This method has been successfully utilized to identify potent inhibitors for targets such as SIRT2, HDAC8, and the ENL YEATS domain.^93–95^ With the advancement of genetic incorporation techniques for methyl- and dimethyl-lysine using amber suppression mutagenesis,^96–99^ combining them with the PADLE technique presents a promising pathway for discovering chromodomain inhibitors. This is an exciting direction we are actively pursuing.

## 5. Concluding remarks

Methyllysine recognizing readers, chromodomains, are integral components of the epigenetic machinery. Binding of chromodomains to methyllysine PTMs facilitates or stabilizes the association of their host proteins at specific PTM-rich genomic sites. This association is required for a wide array of normal cellular processes and is dysregulated in diseases, making chromodomains promising targets for drug discovery. To date, selective inhibitors have been identified for only a limited number of chromodomains, underscoring the need for further research in this direction. Emerging technologies and PROTAC-based approaches can offer exciting avenues exploiting specific ligands to degrade chromodomain-containing proteins.^100–102^ Additionally, peptide display libraries containing peptides with unnatural amino acids, analogous to DNA-encoded libraries, can screen millions of peptides and have already shown promise in creating selective peptide ligands/inhibitors for epigenetic reader proteins.^94,95,103^ Leads from these screens can be further optimized through structure–activity relationship (SAR) studies to develop potent and selective inhibitors. In summary, while the development of chromodomain inhibitors is still in its early stages, this direction represents a dynamic and promising area of research.

## Data availability

This is a review article. It does not contain original scientific data that need to be released.

## Conflicts of interest

The authors declare no competing interests.

## References

[cit1] Strahl B. D., Allis C. D. (2000). Nature.

[cit2] Jenuwein T., Allis C. D. (2001). Science.

[cit3] Kouzarides T. (2007). Cell.

[cit4] Chi P., Allis C. D., Wang G. G. (2010). Nat. Rev. Cancer.

[cit5] Polak P., Karlic R., Koren A., Thurman R., Sandstrom R., Lawrence M. S., Reynolds A., Rynes E., Vlahovicek K., Stamatoyannopoulos J. A., Sunyaev S. R. (2015). Nature.

[cit6] Shen H., Laird P. W. (2013). Cell.

[cit7] Taverna S. D., Li H., Ruthenburg A. J., Allis C. D., Patel D. J. (2007). Nat. Struct. Mol. Biol..

[cit8] Musselman C. A., Lalonde M. E., Cote J., Kutateladze T. G. (2012). Nat. Struct. Mol. Biol..

[cit9] Andrews F. H., Strahl B. D., Kutateladze T. G. (2016). Nat. Chem. Biol..

[cit10] Cochran A. G., Conery A. R., Sims R. J. (2019). Nat. Rev. Drug Discovery.

[cit11] Bannister A. J., Zegerman P., Partridge J. F., Miska E. A., Thomas J. O., Allshire R. C., Kouzarides T. (2001). Nature.

[cit12] Nielsen P. R., Nietlispach D., Mott H. R., Callaghan J., Bannister A., Kouzarides T., Murzin A. G., Murzina N. V., Laue E. D. (2002). Nature.

[cit13] Jacobs S. A., Khorasanizadeh S. (2002). Science.

[cit14] Santiago C., Nguyen K., Schapira M. (2011). J. Comput.-Aided Mol. Des..

[cit15] Paro R., Hogness D. S. (1991). Proc. Natl. Acad. Sci. U. S. A..

[cit16] Maurer-Stroh S., Dickens N. J., Hughes-Davies L., Kouzarides T., Eisenhaber F., Ponting C. P. (2003). Trends Biochem. Sci..

[cit17] Yap K. L., Zhou M. M. (2011). Biochemistry.

[cit18] Blus B. J., Wiggins K., Khorasanizadeh S. (2011). Crit. Rev. Biochem. Mol. Biol..

[cit19] Hou X., Xu M., Zhu C., Gao J., Li M., Chen X., Sun C., Nashan B., Zang J., Zhou Y. (2023). Nat. Commun..

[cit20] Lachner M., O'Carroll D., Rea S., Mechtler K., Jenuwein T. (2001). Nature.

[cit21] Jacobs S. A., Khorasanizadeh S. (2002). Science.

[cit22] Jacobs S. A., Taverna S. D., Zhang Y., Briggs S. D., Li J., Eissenberg J. C., Allis C. D., Khorasanizadeh S. (2001). EMBO J..

[cit23] Nielsen P. R., Nietlispach D., Mott H. R., Callaghan J., Bannister A., Kouzarides T., Murzin A. G., Murzina N. V., Laue E. D. (2002). Nature.

[cit24] Kaustov L., Ouyang H., Amaya M., Lemak A., Nady N., Duan S., Wasney G. A., Li Z., Vedadi M., Schapira M. (2011). J. Biol. Chem..

[cit25] Min J., Zhang Y., Xu R.-M. (2003). Genes Dev..

[cit26] Fischle W., Wang Y., Jacobs S. A., Kim Y., Allis C. D., Khorasanizadeh S. (2003). Genes Dev..

[cit27] Bracken A. P., Helin K. (2009). Nat. Rev. Cancer.

[cit28] Sparmann A., Van Lohuizen M. (2006). Nat. Rev. Cancer.

[cit29] Zheng Z.-Q., Yuan G.-Q., Kang N.-L., Nie Q.-Q., Zhang G.-G., Wang Z. (2022). Front. Neurol..

[cit30] Li J., Xu Z., Zhou L., Hu K. (2022). Aging.

[cit31] Li Q., Fu L., Wu D., Wang J. (2021). J. Gastrointest. Oncol..

[cit32] Iqbal M. A., Siddiqui S., Ur Rehman A., Siddiqui F. A., Singh P., Kumar B., Saluja D. (2021). Mol. Oncol..

[cit33] Xie X., Ning Y., Long J., Wang H., Chen X. (2020). FEBS Open Bio.

[cit34] Ma T., Ma N., Chen J.-L., Tang F.-X., Zong Z., Yu Z.-M., Chen S., Zhou T.-C. (2020). J. Gastrointest. Oncol..

[cit35] Hu K., Yao L., Xu Z., Yan Y., Li J. (2022). Front. Cell Dev. Biol..

[cit36] Mao G., Zheng Y., Lin S., Ma L., Zhou Z., Zhang S. (2021). Int. J. Gen. Med..

[cit37] Zhou J., Chen Z., Zou M., Wan R., Wu T., Luo Y., Wu G., Wang W., Liu T. (2021). Front. Neurol..

[cit38] Zhang X., Zhou W., Zhang Y., Liu Z. (2022). Int. J. Gen. Med..

[cit39] Li D., Liu Y., Hao S., Chen B., Li A. (2020). J. Clin. Lab. Anal..

[cit40] Wang J., Yang B., Zhang X., Liu S., Pan X., Ma C., Ma S., Yu D., Wu W. (2023). Int. J. Oncol..

[cit41] Flanagan J. F., Mi L.-Z., Chruszcz M., Cymborowski M., Clines K. L., Kim Y., Minor W., Rastinejad F., Khorasanizadeh S. (2005). Nature.

[cit42] Mills A. A. (2017). Cold Spring Harbor Perspect. Med..

[cit43] Lang J., Tobias E., Mackie R. (2011). Br. J. Dermatol..

[cit44] Zhao R., Meng F., Wang N., Ma W., Yan Q. (2014). PLoS One.

[cit45] Wang L., He S., Yanyang T., Ji P., Zong J., Zhang J., Feng F., Zhao J., Gao G., Zhang Y. (2013). J. Clin. Neurosci..

[cit46] Bagchi A., Papazoglu C., Wu Y., Capurso D., Brodt M., Francis D., Bredel M., Vogel H., Mills A. A. (2007). Cell.

[cit47] Zhao R., Yan Q., Lv J., Huang H., Zheng W., Zhang B., Ma W. (2012). Lung Cancer.

[cit48] Robbins C. M., Tembe W. A., Baker A., Sinari S., Moses T. Y., Beckstrom-Sternberg S., Beckstrom-Sternberg J., Barrett M., Long J., Chinnaiyan A. (2011). Genome Res..

[cit49] Wu X., Zhu Z., Li W., Fu X., Su D., Fu L., Zhang Z., Luo A., Sun X., Fu L. (2012). Breast Cancer Res..

[cit50] Mulero-Navarro S., Esteller M. (2008). Epigenetics.

[cit51] Garcia I., Mayol G., Rodríguez E., Suñol M., Gershon T. R., Ríos J., Cheung N.-K. V., Kieran M. W., George R. E., Perez-Atayde A. R. (2010). Mol. Cancer.

[cit52] Wang J., Chen H., Fu S., Xu Z.-M., Sun K.-L., Fu W.-N. (2011). Oral Oncol..

[cit53] Guillemette S., Serra R. W., Peng M., Hayes J. A., Konstantinopoulos P. A., Green M. R., Cantor S. B. (2015). Genes Dev..

[cit54] Russo J., Russo I. H. (2012). Horm. Mol. Biol. Clin. Invest..

[cit55] Brajadenta G. S., Bilan F., Gilbert-Dussardier B., Kitzis A., Thoreau V. (2019). Eur. J. Hum. Genet..

[cit56] Pagon R. A., Graham J. M., Zonana J., Yong S.-L. (1981). J. Pediatr..

[cit57] Layman W. S., Hurd E. A., Martin D. M. (2010). Clin. Genet..

[cit58] Lee J. M., Lee J. S., Kim H., Kim K., Park H., Kim J.-Y., Lee S. H., Kim I. S., Kim J., Lee M. (2012). Mol. Cell.

[cit59] Kokura K., Sun L., Bedford M. T., Fang J. (2010). EMBO J..

[cit60] Sun L., Kokura K., Izumi V., Koomen J. M., Seto E., Chen J., Fang J. (2015). EMBO Rep..

[cit61] Zhang N., Chi M., Pan W., Zhang C., Wang Y., Gao X., Bai C., Liu X. (2024). Oncol. Lett..

[cit62] Wang Y., Xiao H., Wang C., Wu H., He H., Yao C., Cui J., Li W. (2020). J. Cell. Biochem..

[cit63] Gu Z., Liu Y., Zhang Y., Cao H., Lyu J., Wang X., Wylie A., Newkirk S. J., Jones A. E., Lee M. (2021). Nat. Genet..

[cit64] Kim J., Daniel J., Espejo A., Lake A., Krishna M., Xia L., Zhang Y., Bedford M. T. (2006). EMBO Rep..

[cit65] Lahn B. T., Tang Z. L., Zhou J., Barndt R. J., Parvinen M., Allis C. D., Page D. C. (2002). Proc. Natl. Acad. Sci. U. S. A..

[cit66] Caron C., Pivot-Pajot C., van Grunsven L. A., Col E., Lestrat C., Rousseaux S., Khochbin S. (2003). EMBO Rep..

[cit67] Arrowsmith C. H., Bountra C., Fish P. V., Lee K., Schapira M. (2012). Nat. Rev. Drug Discovery.

[cit68] Ortiz G., Kutateladze T. G., Fujimori D. G. (2023). Curr. Opin. Chem. Biol..

[cit69] Musselman C. A., Khorasanizadeh S., Kutateladze T. G. (1839). Biochem. Biophys. Acta.

[cit70] Simhadri C., Daze K. D., Douglas S. F., Quon T. T., Dev A., Gignac M. C., Peng F., Heller M., Boulanger M. J., Wulff J. E. (2014). J. Med. Chem..

[cit71] Simhadri C., Gignac M. C., Anderson C. J., Milosevich N., Dheri A., Prashar N., Flemmer R. T., Dev A., Henderson T. G., Douglas S. F. (2016). ACS Omega.

[cit72] Stuckey J. I., Dickson B. M., Cheng N., Liu Y., Norris J. L., Cholensky S. H., Tempel W., Qin S., Huber K. G., Sagum C. (2016). Nat. Chem. Biol..

[cit73] Stuckey J. I., Simpson C., Norris-Drouin J. L., Cholensky S. H., Lee J., Pasca R., Cheng N., Dickson B. M., Pearce K. H., Frye S. V. (2016). J. Med. Chem..

[cit74] Lamb K. N., Bsteh D., Dishman S. N., Moussa H. F., Fan H., Stuckey J. I., Norris J. L., Cholensky S. H., Li D., Wang J. (2019). Cell Chem. Biol..

[cit75] Ren C., Morohashi K., Plotnikov A. N., Jakoncic J., Smith S. G., Li J., Zeng L., Rodriguez Y., Stojanoff V., Walsh M. (2015). Chem. Biol..

[cit76] Ren C., Smith S. G., Yap K., Li S., Li J., Mezei M., Rodriguez Y., Vincek A., Aguilo F., Walsh M. J. (2016). ACS Med. Chem. Lett..

[cit77] Milosevich N., Gignac M. C., McFarlane J., Simhadri C., Horvath S., Daze K. D., Croft C. S., Dheri A., Quon T. T., Douglas S. F. (2016). ACS Med. Chem. Lett..

[cit78] Milosevich N., Wilson C. R., Brown T. M., Alpsoy A., Wang S., Connelly K. E., Sinclair K. A., Ponio F. R., Hof R., Dykhuizen E. C. (2021). ChemMedChem.

[cit79] Wang S., Denton K. E., Hobbs K. F., Weaver T., McFarlane J. M., Connelly K. E., Gignac M. C., Milosevich N., Hof F., Paci I. (2019). ACS Chem. Biol..

[cit80] Suh J. L., Bsteh D., Hart B., Si Y., Weaver T. M., Pribitzer C., Lau R., Soni S., Ogana H., Rectenwald J. M. (2022). Cell Chem. Biol..

[cit81] Wang S., Alpsoy A., Sood S., Ordonez-Rubiano S. C., Dhiman A., Sun Y., Jiao G., Krusemark C. J., Dykhuizen E. C. (2021). ChemBioChem.

[cit82] Lercher L., Simon N., Bergmann A., Tauchert M., Bochmann D., Bashir T., Neuefeind T., Riley D., Danna B., Krawczuk P. (2022). Slas Discovery.

[cit83] Lamb K. N., Dishman S. N., Waybright J. M., Engelberg I. A., Rectenwald J. M., Norris-Drouin J. L., Cholensky S. H., Pearce K. H., James L. I., Frye S. V. (2021). ACS Omega.

[cit84] Johnson R. L., Graboski A. L., Li F., Norris-Drouin J. L., Walton W. G., Arrowsmith C. H., Redinbo M. R., Frye S. V., James L. I. (2024). J. Med. Chem..

[cit85] Barnash K., Lamb K., Stuckey J., Norris J., Cholensky S., Kireev D., Frye S., James L. (2016). ACS Chem. Biol..

[cit86] Dong C., Liu Y., Lyu T.-J., Beldar S., Lamb K. N., Tempel W., Li Y., Li Z., James L. I., Qin S. (2020). Cell Chem. Biol..

[cit87] Waybright J. M., Clinkscales S. E., Barnash K. D., Budziszewski G. R., Rectenwald J. M., Chiarella A. M., Norris-Drouin J. L., Cholensky S. H., Pearce K. H., Herring L. E. (2021). ACS Chem. Biol..

[cit88] Clermont P.-L., Lin D., Crea F., Wu R., Xue H., Wang Y., Thu K. L., Lam W. L., Collins C. C., Wang Y. (2015). Clin. Epigen..

[cit89] Kean K. M., Baril S. A., Lamb K. N., Dishman S. N., Treacy J. W., Houk K. N., Brustad E. M., James L. I., Waters M. L. (2022). J. Med. Chem..

[cit90] Fan Y., Feng R., Zhang X., Wang Z. L., Xiong F., Zhang S., Zhong Z. F., Yu H., Zhang Q. W., Zhang Z., Wang Y., Li G. (2024). Acta Pharm. Sin. B.

[cit91] Veggiani G., Villasenor R., Martyn G. D., Tang J. Q., Krone M. W., Gu J., Chen C., Waters M. L., Pearce K. H., Baubec T., Sidhu S. S. (2022). Nat. Commun..

[cit92] Tharp J. M., Hampton J. T., Reed C. A., Ehnbom A., Chen P. C., Morse J. S., Kurra Y., Perez L. M., Xu S., Liu W. R. (2020). Nat. Commun..

[cit93] Morse J. S., Sheng Y. J., Hampton J. T., Sylvain L. D., Das S., Alugubelli Y. R., Chen P. C., Yang K. S., Xu S., Fierke C. A., Liu W. R. (2022). Protein Sci..

[cit94] Chen P.-H. C., Guo X. S., Zhang H. E., Dubey G. K., Geng Z. Z., Fierke C. A., Xu S., Hampton J. T., Liu W. R. (2024). ACS Cent. Sci..

[cit95] Tharp J. M., Hampton J. T., Reed C. A., Ehnbom A., Chen P.-H. C., Morse J. S., Kurra Y., Pérez L. M., Xu S., Liu W. R. (2020). Nat. Commun..

[cit96] Wang Y. S., Wu B., Wang Z., Huang Y., Wan W., Russell W. K., Pai P. J., Moe Y. N., Russell D. H., Liu W. R. (2010). Mol. BioSyst..

[cit97] Wang Z. A., Zeng Y., Kurra Y., Wang X., Tharp J. M., Vatansever E. C., Hsu W. W., Dai S., Fang X., Liu W. R. (2017). Angew. Chem., Int. Ed..

[cit98] Nguyen D. P., Garcia Alai M. M., Kapadnis P. B., Neumann H., Chin J. W. (2009). J. Am. Chem. Soc..

[cit99] Nguyen D. P., Garcia Alai M. M., Virdee S., Chin J. W. (2010). Chem. Biol..

[cit100] Kong Y., Lan T., Wang L., Gong C., Lv W., Zhang H., Zhou C., Sun X., Liu W., Huang H. (2024). Oncogene.

[cit101] Zoppi V., Hughes S. J., Maniaci C., Testa A., Gmaschitz T., Wieshofer C., Koegl M., Riching K. M., Daniels D. L., Spallarossa A. (2018). J. Med. Chem..

[cit102] Wang C., Zhang Y., Yang S., Chen W., Xing D. (2022). J. Enzyme Inhib. Med. Chem..

[cit103] Low J. K., Patel K., Jones N., Solomon P., Norman A., Maxwell J. W., Pachl P., Matthews J. M., Payne R. J., Passioura T. (2023). J. Biol. Chem..

